# *Notes from the Field*: Death of a Farm Worker After Exposure to Manure Gas in an Open Air Environment — Wisconsin, August 2016

**DOI:** 10.15585/mmwr.mm6632a6

**Published:** 2017-08-18

**Authors:** John M. Shutske, Rebecca A. Larson, Daniel M. Schaefer, Liz Y. Binversie, Scott Rifleman, Cheryl Skjolaas

**Affiliations:** ^1^Department of Biological Systems Engineering, College of Agricultural and Life Sciences, University of Wisconsin, Madison; ^2^Department of Animal Science, College of Agricultural and Life Sciences, University of Wisconsin, Madison; ^4^University of Wisconsin-Extension, Brown County; ^5^Portage County Wisconsin Coroner’s Office.

On August 15, 2016, at approximately 6:30 a.m., a previously healthy male employee of a Wisconsin beef farm was found dead near the edge of an outdoor 60,400 square foot (1.4 acre) manure storage basin (Figure). The basin was approximately 15 feet (4.6 meters) deep and nearly full. The victim, aged 29 years, was discovered by another worker; the coroner was notified at 6:50 a.m., and he pronounced the victim dead at the scene. Thirteen dead cattle were discovered in an adjoining pen; three others were struggling to stand and were euthanized. The owner of the farm reported that at 3:00 a.m., the victim had used a tractor-powered agitator to agitate the manure,* which a contractor was scheduled to pump and spread on cropland later that morning. The last contact from the victim was a social media post at 4:10 a.m. At the time he was discovered, he was approximately 3 feet downslope from the rear of the tractor, which was running.

**FIGURE:**
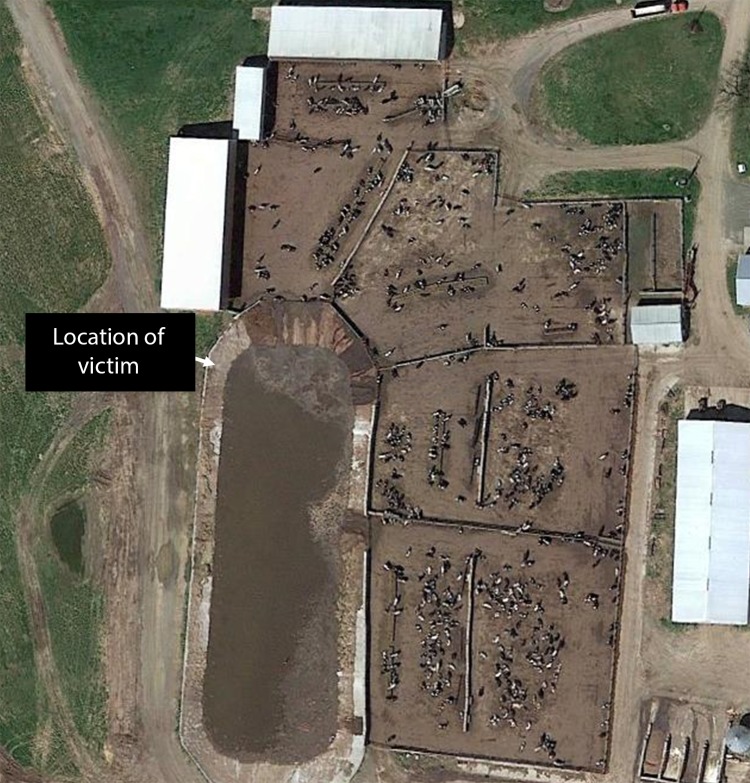
Open-air manure pit and **s**ite of death of a worker on a beef farm — Wisconsin, August 2016 **Source:** Google Earth.

Weather conditions from a nearby airport reported temperatures at 4:15 a.m., 5:15 a.m., and 6:15 a.m. of 54.5°F (12.5°C), 53.6°F (12.0°C), and 52.9°F (11.6°C), respectively, with no wind. The high temperature the previous day was 80°F (26.7°C), and reached 87°F (30.6°C) the preceding week (August 7–13), which was 10°F (5.6°C) warmer than the historical weekly average. Relative humidity measured at the nearby airport during these same time intervals ranged from 97% to 100%. The National Weather Service’s Green Bay office documented a temperature inversion in the area that morning, citing warmer air temperatures 1,000–1,300 feet (300–400 meters) above ground level.

The man’s death was initially attributed to methane, a physiologically inert gas produced through anaerobic decomposition of organic matter in manure and released through liquid manure. Methane deaths are usually the result of asphyxiation (*1*). The coroner reported foam coming from the decedent’s mouth and nose, suggesting pulmonary edema; there was no indication of external trauma, and an autopsy was not conducted. A University of Wisconsin farm safety expert advised the coroner to test the decedent’s blood for evidence of hydrogen sulfide exposure; blood thiosulfate level was 9.2 *μ*g/mL, consistent with lethal hydrogen sulfide exposure as the cause of death (*2*). The cattle deaths were also assumed to have resulted from hydrogen sulfide exposure, although this was not laboratory-confirmed.

University staff members visited the farm on September 26, 2016, to ascertain potential sources of sulfur that might have caused elevated hydrogen sulfide levels in the stored manure, such as gypsum animal bedding (*3*). Ambient air was not tested, because no agitation was occurring at the time of the visit, and weather conditions were considerably different than they had been on the day of the event. Although no gypsum was used, the animals’ diet did include distiller’s syrup, a by-product of corn-based ethanol production. The sulfur concentration in a tested syrup sample (collected the day of the visit, stored in a refrigerator, and tested on January 20, 2017) was 1.53% of dry matter; 18–20 pounds of syrup were fed per day to each animal. At the recommendation of a cattle nutritionist, the farmer was providing thiamine supplementation to prevent polioencephalomalacia, a neurologic disease of ruminants that has been associated with thiamine status and high sulfur intake (*4*). Previous laboratory tests of the herd’s mixed feed analyzed on September 16, 2016, found a sulfur concentration of 0.44% of diet dry matter. Cattle nutrition references recommend that for feedlot cattle, the maximum tolerable limit for dietary sulfur is 0.3% of diet dry matter, with 0.15% considered sufficient (*5*).

Manure tested twice during the previous year had sulfur levels of 9.67 and 6.94 pounds per thousand gallons for samples tested on April 15, 2015, and November 9, 2015, respectively. No additional manure samples were taken immediately before or after the incident. The average manure sulfur level for Wisconsin beef operations is 1.6 pounds per thousand gallons (*6*).

Asphyxiation deaths associated with manure storage typically occur in confined spaces not intended for continuous occupancy (*1*). This incident was unusual because human and cattle deaths occurred in an outdoor, ambient air environment. It is possible that the temperature inversion and zero wind velocity suppressed air mixing, leading to an accumulation of lethal concentrations of hydrogen sulfide at ground level as agitation occurred.^†^ Additional research on the impact of weather and other environmental conditions on outdoor gas dispersion, as well as production practices that increase hydrogen sulfide exposure risk is needed. Monitoring for toxic gases and adequate oxygen is important even near outdoor manure storage sites. Improved understanding of factors that contribute to toxic outdoor hydrogen sulfide concentrations is needed to develop worker safety recommendations and to inform outdoor air monitoring strategies. Public health officials and forensic toxicologists who evaluate manure gas incidents should always consider tests for hydrogen sulfide exposure. Farm owners, operators, and employees, as well as professional and volunteer responders in rural areas, should receive additional manure gas education that includes information about hydrogen sulfide, other lethal gases, and the production practices and conditions that increase risk.

## Footnotes

* This agitator was attached to a tractor and driven by a rotating shaft exiting the rear of the tractor. The agitator extended approximately 40 feet (12 meters) outward into the liquid slurry. Agitation action includes a large, rotating propeller that stirs aggressively. The agitator also pumps and sprays the thick liquid backward or to the side to move the manure and break up crusted surfaces.

† Occupational Safety and Health Administration documents lethal concentrations to be 700–1000 ppm with “rapid unconsciousness, ‘knockdown’ or immediate collapse within 1 to 2 breaths, breathing stops, death within minutes.” (https://www.osha.gov/SLTC/hydrogensulfide/hazards.html.) In addition, the relative gas density of hydrogen sulfide is 1.19 (https://www.cdc.gov/niosh/npg/npgd0337.html); hydrogen sulfide gas is heavier than air, so the gas being released during agitation was less likely to be dispersed and remained close to the ground surface.
